# Learning to learn to expand freedom in choices

**DOI:** 10.3389/fpsyg.2013.00780

**Published:** 2013-10-25

**Authors:** Carine Signoret

**Affiliations:** ^1^Linnaeus Centre for Hearing and Deafness (HEAD), Swedish Institute for Disability Research (SIDR)Linköping, Sweden; ^2^Disability Research Division, Department of Behavioural Science and Learning, Linköping UniversityLinköping, Sweden

**Keywords:** teaching-learning activities, knowledge, students, career choice, teacher education, autonomy support

“Once an idea has taken hold of the brain it's almost impossible to eradicate. An idea that is fully formed, fully understood. That sticks, right in there somewhere. *[he points to his head]*” (Inception, 2010).

## Introduction

Learning is related to knowledge that is shared between teacher and students. Whatever the use of traditional or modern teaching-learning methods, the students learn: they have access to new information and can therefore acquire or modify knowledge stored in memory. The goal of this opinion paper is to present what we know about the consequences of this internal change and how this could affect the students' choices. Recent works concerning the influence of knowledge stored in long-term memory (LTM) on the perception of the environment highlight that acquired knowledge could directly and automatically influence our perception of the events in the environment. Indeed, perception is therefore based on acquired knowledge: basically, we perceive what we already know. It is now of the utmost importance to ask how teaching-learning activities chosen by teachers or universities influence the knowledge acquisition and what the consequences of learning are for students' choices.

## On the definition of learning

Learning is a process that enables the integration of information from the environment into memory. That is the main activity of our brain, constantly changing its structure to take into account the encountered experiences, even for adults (Gilkey and Kilts, 2007). Learning begins with sensory perception of some events of our environment, continues with the processing of the perceived events in working-memory, and ends with the storage in LTM of the processed information (Eustache and Desgranges, 2008; Baddeley, 2010).

Sensory memory is an automatic memory, the fruit of our perceptual abilities, usually fading in less than a second. The sensory memory is modality-dependent: we talk about the iconic memory of visual perception and the echoic memory of sound perception (Fietta and Fietta, 2011). Working-memory is the cognitive process that allows the manipulation of perceived information that is currently being used. It involves processes of reasoning, such as reading, writing or calculating (Baddeley, 2012). Working-memory is composed of several independent systems, which implies that we are not aware of all information that is stored there at any given time. For example, when we drive a car, we perform several complex tasks simultaneously. The processed information is then stored in LTM as a function of its nature (Tulving, 1985; Henke, 2010). The LTM includes recent memory, where memories are still fragile, and the memory of past actions that have been consolidated (e.g., by repetition).

Learning can occur after three major basic processes: encoding, storage and information retrieval. When we perceive something, our brain encodes the different features of the target (e.g., shape, color, smell, sound, place) in different neural assemblies (Crick and Koch, 1990; Engel et al., 1999). It is the relationship between these neuronal assemblies, distributed in different places in the brain, which allows a conscious perception of the target. The encoded information is then temporarily stored in working-memory to be compared with knowledge stored in LTM (Baddeley, 2000). When the encoded information is similar to that stored in LTM, processing is rapid and implicit. However, when the encoded information has no representation in LTM, processing becomes slower and explicit, and additional cognitive resources are required in working-memory (Rönnberg et al., 2013). Finally, for recovering information stored in LTM, similar cognitive mechanisms to the ones used during the encoding phase are required: we must rebuild the relationships between the different neural assemblies to remember the encoded target (Nyberg et al., 1996; Crick and Koch, 2003; Hofer et al., 2007). The recovery of the information stored in LTM is traditionally divided into two types. Recalling an item from LTM involves an active return of information by the whole neurons assembly involved in the memory of a target. By contrast, recognition requires a partial activation triggered by a portion of the neurons assembly, which may be sufficient to enable the entire network in the case of recognition.

We now understand that learning is effective when students are able to use information stored in LTM. But what do we know about the influence of the successfully stored information?

## On the influence of knowledge

Knowledge stored in memory could have an influence not only on subsequent processing of the perceived information but also directly on our perception of the events present in our environment. This influence could be observed at a behavioral, but also neurological level.

The knowledge influence on the processing of the perceived information has been demonstrated with the priming paradigm (Neely, 1977). The idea, developed by cognitive psychologists, is to present two stimuli successively, the first called *prime* and the second called *target*. Participants are engaged in a task on the second stimulus, which is usually a lexical-decision task (i.e., to decide if the target is a word or not), but no task is required on the prime. Participant performance (correct responses and reaction times) is better in conditions where the prime and the target are related (e.g., semantic relationship) than unrelated. This is a concrete example of the influence of knowledge stored in LTM on the processing of incoming information present in the environment. This influence has been observed in different modalities (Holcomb and Neville, 1990) and occurs even if the prime is not consciously perceived (Van den Bussche et al., 2009; Daltrozzo et al., 2011). At a neural level, the priming effects are reflected by the *N400 effect*: by investigating the priming effects with electroencephalographic recordings, Kutas and Hillyard (1980) have shown that the brain is able to detect whether the two stimuli are related or not after around 400 ms after the beginning of the target.

Another example of the knowledge influence on the processing of perceived information has been provided with the *pop-out effect* (Davis et al., 2005). Giving participants the opportunity to hear an unintelligible degraded sentence after they have been informed about its content, produces a subjectively greater perceptual clarity of the degraded sentences. In other words, the first time participants heard the degraded sentence, they perceived noise, but once they knew what the content of the degraded sentence was, they could hear each word of the sentence that was subjectively perceived as less noisy (Wild et al., 2012). The pop-out effect probably arises when the auditory system is able to match auditory input with top-down predictions of knowledge processed in working memory. The predictions established in the brain could help to explain the incoming information (a similar phenomenon has been described for visual object perception, see Kersten et al., 2004). The pop-out effect seems then to provide evidence that knowledge stored in memory predicts speech perception (Sohoglu et al., 2012).

The influence of knowledge stored in memory has also been demonstrated on the first level of perception, which is the detection of the events in the environment. In a first study presenting different types of sounds at several intensity levels (Signoret et al., 2011), the results showed that participants obtained better detection performance for speech sounds than for complex sounds (Speech Detection Effect), and better detection performance for words than for pseudo-words and complex sounds (Word Detection Effect), when the detection becomes difficult (around the perception threshold). These results suggest that phonological and semantic knowledge improve the auditory detection of sounds. In a second study investigating the neural correlates of speech perception using electroencephalographic recordings (Signoret et al., 2013), participants were asked to categorize different types of sounds. The results showed that the amplitude of the N1 component elicited in the sensory cortex was smaller in response to words than in response to pseudo-words, and also smaller in response of pseudo-words than in response to complex sounds. These results suggest then that the brain is influenced by knowledge stored in memory from 100 ms after the beginning of the presentation of the stimuli to be detected. Current research is investigating if the influence of knowledge could be observed even before the presentation of the event in the environment (Arnal and Giraud, 2012), constraining the brain and even the brainstem (Sörqvist et al., 2012) to process preferentially expected stimuli.

We better detect events from the environment for which we already have representations stored in memory (a word of our language for instance) than events from the environment for which we have no representation stored in memory (a pseudo-word, or a noise as complex as speech). The most likely explanation of these results is that the brain acts predictively using knowledge stored in memory, even before the occurrence of information in the environment, constraining the processing of the incoming information. The stage is set now for asking the question about the influence of knowledge that the student is learning in their choices (Bargh et al., 2012).

## Consequences for learning

In a systematic review (Stagg et al., 2012), it has been shown that the teaching style, from as little as three weeks' duration of exposure, influences the career choice of students when the teacher was rated as a high quality teacher. The longer the exposure to high-quality teaching, the greater the influence on student career choices. However, when students judged a teacher as being a negative role model, a poor teacher or lacking discipline-specific knowledge, they will turn away from that field.

Despite the fact they have received the same amount of information, the students develop better learning during the courses with a teacher rated as a high quality teacher than with a teacher rated as a poor quality teacher (Wayne and Youngs, 2003). We can then speculate that if the learning was better, the knowledge stored in LTM has a better quality: better encoding, better consolidation and better recovery. The students could experience the feeling of understanding, access the knowledge, and then use the knowledge stored in memory in new situations. When the time of the career choice comes, it could be argued that it would be easier for the students to choose the career of which they have the better knowledge. Regarding behavioral and neural results reported on the influence of knowledge on the first step of perception (i.e., the detection), it might be also possible that the students have even not detected the career propositions for which they had bad or less knowledge. Indeed, it could be suggested that the knowledge stored in memory is not of sufficient quality to be usable when required. It can then be reasonably suggested that the students will not choose a career for which they feel they have little or no knowledge.

This example is only one illustration of the degree of influence of teaching/learning on the students' choices. If the information for which we already have representation in memory is better perceived, this means that the information for which we already have representation in memory will be preferentially processed, in an automatic and unconscious fashion. This way it becomes difficult to process totally new information, such as trying to learn a new language that is not based on a known alphabet for instance. It is like we become prisoners of our knowledge each time we need to use knowledge stored in LTM. And we know that we are always referring to our knowledge stored in memory for processing incoming events (Signoret et al., 2011), decision-making, moral judgments, emotional processes, face perception and social judgment (Bargh et al., 2012).

Teachers should be aware of the influence of the learning contents on students' choices. Teachers are supposed to want the students to acquire a maximum amount of knowledge to expand their freedom to use the appropriated knowledge. However, the knowledge field is incommensurable and nobody is able to know, and consequently, to learn everything. One possible solution for avoiding this limitation in learning is related to the learning autonomy of students. Learning to learn is the key solution for surpassing this limitation and expanding freedom of mind. Reported as a virtue of the 21st century (Deakin Crick and Wilson, 2005), the learning to learn ability is involved in choices throughout the lifespan (Hoskins and Deakin Crick, 2010) and is reported as an indicator of adaptability in response to everyday challenges (Deakin Crick et al., 2013). The students will not refer to their knowledge before making a choice, but they will be able to find and to acquire the knowledge useful for making that choice. Learning to learn to develop interest and curiosity of the students should be a central point in the student-based educational approach (Black et al., 2006), which could be developed using the problem-based learning method for example. The implications for teacher education training are that the needs and competency of teachers in promoting self-determination should be examined (Hui and Tsang, 2012) in order to acquire knowledge and skills to encourage an autonomy-supportive classroom environment and facilitate students to be self-regulated learners.
